# Multi-analyte biosensor interface for real-time monitoring of 3D microtissue spheroids in hanging-drop networks

**DOI:** 10.1038/micronano.2016.22

**Published:** 2016-06-06

**Authors:** Patrick M. Misun, Jörg Rothe, Yannick R.F. Schmid, Andreas Hierlemann, Olivier Frey

**Affiliations:** 1 ETH Zürich, Department of Biosystems Science and Engineering, Bio Engineering Laboratory, Mattenstrasse 26, Basel CH-4058, Switzerland

**Keywords:** biosensor, body on a chip, glucose, hanging drop, lactate, metabolism, spheroid

## Abstract

Microfluidics is becoming a technology of growing interest for building microphysiological systems with integrated read-out functionalities. Here we present the integration of enzyme-based multi-analyte biosensors into a multi-tissue culture platform for ‘body-on-a-chip’ applications. The microfluidic platform is based on the technology of hanging-drop networks, which is designed for the formation, cultivation, and analysis of fluidically interconnected organotypic spherical three-dimensional (3D) microtissues of multiple cell types. The sensor modules were designed as small glass plug-ins featuring four platinum working electrodes, a platinum counter electrode, and an Ag/AgCl reference electrode. They were placed directly into the ceiling substrate from which the hanging drops that host the spheroid cultures are suspended. The electrodes were functionalized with oxidase enzymes to enable continuous monitoring of lactate and glucose through amperometry. The biosensors featured high sensitivities of 322±41 nA mM^−1^ mm^−2^ for glucose and 443±37 nA mM^−1^ mm^−2^ for lactate; the corresponding limits of detection were below 10 μM. The proposed technology enabled tissue-size-dependent, real-time detection of lactate secretion from single human colon cancer microtissues cultured in the hanging drops. Furthermore, glucose consumption and lactate secretion were monitored in parallel, and the impact of different culture conditions on the metabolism of cancer microtissues was recorded in real-time.

## Introduction

Microsystems technology offers a variety of new approaches to culturing and analyzing human cells and functional tissue structures^1,2^. Tissue models that reproduce *in vivo* conditions as closely as possible are key for understanding organ-specific cell behavior, for investigating diseases, and for discovering new agents and therapies during the drug-development process^3–6^.

Simple organ models for testing new compounds and studying cellular response and metabolism in preclinical trials consist of two-dimensional (2D) cell culture systems. Culturing cells in a monolayer has certain advantages; the cells are simple to handle and image, and one can rely on established techniques and standard laboratory equipment. Two-dimensional cell cultures, however, have certain limitations in mimicking functional living tissue, as original microenvironmental inputs, such as cell-to-cell interaction and spatiotemporal biochemical gradients, are absent^7–9^. Three-dimensional (3D) cell cultures offer the potential to overcome many of these limitations and are therefore finding increasing applications in the pharmaceutical industry and basic research^10^. A wide spectrum of 3D cell culture methods and materials are currently being developed to reproduce tissue-specific properties^11–13^. The hanging-drop technique, for example, is a popular method of growing scaffold-free 3D microtissues of spherical shape. Different types of spheroids can be formed to mimic the functional tissues of various organs while retaining their specific characteristics and functions over an extended period of time^14–19^.

Two additional aspects are closely related and of high interest. The first is that advanced culture systems with continuous perfusion capabilities and the possibility to integrate fluid-dynamic and mechanodynamic cues offer the possibility to even better mimic the *in vivo* environment^20^. Perfusion, for example, can be modulated to provide tissue-specific environmental conditions^21,22^ and to simulate the shear forces that are present at the interfaces of tissues in their fluidic environments^23–25^. Flexible substrates can be implemented to mimic the oscillatory mechanical stress in lung aveoli^26,27^. Finally, interconnected fluidic networks offer the possibility to combine different cell cultures or tissue types to realize ‘body-on-a-chip’ configurations^28–34^.

The second aspect is related to established analysis methods, which have been designed primarily to meet the requirements of 2D cell cultures. These analysis methods and assays must be adapted for application to 3D cell cultures and the corresponding platforms to fully capitalize on the advantages of 3D cell culture formats.

Most metabolic processes are dynamic and occur on time scales of a few minutes. Thus, studying time-resolved responses of cell cultures to environmental changes or upon the application of a defined compound dosage often requires continuous read-out to ensure that occurrences of important events are not missed^35^. In well-based approaches, liquid handling is discrete, which engenders a high risk of missing events. Moreover, conventional cell assays usually involve rather large sample volumes. Frequent sampling of the cell culture medium interrupts and disturbs the overall culturing process, increases the risk of contamination, and may cause extensive dilution of important markers or metabolites in the process because of the addition of new medium. As the cell-to-medium ratios are extremely low, frequent sampling also incurs a risk that metabolite concentrations may fall below the limit of detection.

Microfluidics, in contrast, offer precise flow handling of small liquid volumes and accurate control over microenvironmental parameters that are important in analytical cell culture devices^1^. In addition, microfabrication techniques offer multiplexing capabilities and allow straightforward integration of novel modules, such as pumping systems, actuators, and microsensors^36,37^. Microsensor technologies have been developed over the past several decades for a variety of applications. Their miniature size and versatile features as well as their high sensitivity and low-detection limits enable monitoring of various analytes in cell and tissue culture set-ups at high temporal and spatial resolution.

Many multi-sensor systems were designed as probes, mostly comprising electrochemical biosensors, and have been applied to conventional cell culture assays to monitor microphysiological conditions over time^38–41^. Miniaturized versions have been used as scanning probes to monitor the glucose and lactate metabolism of single cells^42,43^. Flow-through-type sensor systems have been devised to analyze cell culture conditions and metabolites in liquid samples downstream of cell cultures^44–51^. Silicon-based sensor chips for multi-parameter online monitoring have been mounted in a perfused cell culture unit^52^, and multiple sensing electrodes have been directly incorporated into transparent microfluidic systems to measure the glucose consumption of single cardiac cells^53^ or the lactate production of cells of various types^54^ and, combined with optical read-out, for monitoring cancer cell metabolism^55^.

The combination and integration of microfluidics and microsensors with cell culturing units poses challenges. The fabrication methods for the different components need to be compatible, and all target features and functions must be preserved over the duration of an experiment; the cell cultures must remain viable and functional, the microfluidic handling needs to be robust and precise, and the sensors must satisfy the detection specifications, including sensitivity and selectivity. The use of 3D microtissues in microfluidic set-ups and the realization of multi-tissue configurations render this integration even more complex.

In this paper, we present a highly versatile, modular, and scalable analytical platform technology that combines microfluidic hanging-drop networks with multi-analyte biosensors for the *in situ* monitoring of 3D microtissue metabolism. Hanging-drop networks consist of arrays of interconnected hanging drops that are specifically designed for 3D microtissue aggregation and culture^56^. They allow precise control of microtissue culture conditions and enable on-chip intertissue communication, which is of fundamental importance for realizing 3D microtissue body-on-a-chip configurations. Sensor modules were implemented as small glass plug-ins to allow convenient functionalization and calibration of the sensors and to avoid interference with microfluidic functions. We demonstrated the detection of lactate secreted by single-microtissue spheroids, the amount of which depends on the tissue size.

Moreover, we were able to monitor the variations in lactate secretion with changing nutrient availability by applying different microfluidic perfusion protocols. Finally, we also showed that it is possible to monitor microtissue lactate secretion and glucose consumption in parallel.

## Materials and methods

### Microfluidic hanging-drop network fabrication

A microfabricated SU-8 master mould was used to fabricate microfluidic polydimethylsiloxane (PDMS) chips (Supplementary Figure S1). Three layers of SU-8 100 (Microchem Corp., Newton, MA, USA) were successively spin-coated onto a 4-inch silicon wafer and processed using a standard photolithographic protocol. The rotation speeds were adjusted to obtain the desired layer thicknesses of *h*_1_=250 μm, *h*_2_=250 μm, and *h*_3_=500 μm. The first two layers were used to pattern the rim and drop structures. The third layer was used to create a recess in the microfluidic PDMS chip architecture for the insertion of the sensor glass plug-in during device assembly.

After soft baking, each SU-8 layer was exposed to ultraviolet light through different transparency masks and post-exposure baked for cross-linking. Development was performed at the end of the fabrication procedure for all SU-8 layers simultaneously. A vapor silanization process with trichloro(1H,1H,2H,2H,-perfluorooctyl)silane (Sigma-Aldrich, Buchs, Switzerland) was used to reduce the adhesion of the PDMS during casting.

The PDMS was prepared by mixing Sylgard 184 Silicone Elastomer and Sylgard 184 Silicon Elastomer Curing Agent (Dow Corning GmbH, Wiesbaden, Germany) at a 10:1 (w/w) ratio. The mixture was then poured onto the SU-8 master mould, degassed for 1 h and cured on a hotplate for 2 h at 80 °C. The cured 3-mm-thick PDMS replica was cut into individual microfluidic chips, and holes were punched at predefined access sites to enable fluidic connections via tubing. Microscopy slides (26 mm×76 mm×1 mm) were prepared with liquid access holes, and the PDMS chips were bonded to these slides after 25 s of oxygen plasma surface activation (Harrick Plasma PDC-002, Harrick Plasma, Ithaca, NY, USA). The glass slide improves the planarity and mechanical stability of the system. Finally, the microfluidic PDMS chips were sterilized with 70% ethanol and isopropanol and cleaned with oxygen plasma for a duration of 3 min (Diener Electronic GmbH & Co., Ebhausen, Germany).

### Sensor glass plug-in fabrication

A 4-inch glass wafer (0.5-mm thick) was used to fabricate the sensor glass plug-ins using standard photolithography processes (Supplementary Figure S2). Lift-off resist (LOR3B, Microchem Corp., Newton, USA) and positive photoresist (S1813, Rohm-Haas, Schwalbach, Germany) were sequentially spin-coated and soft-baked to form layers with thicknesses of 300–400 nm and 1.5 μm, respectively. Metal patterns were transferred into the resist via ultraviolet exposure through a transparency mask. The wafer was developed in MF319 Developer (Rohm-Haas, Schwalbach, Germany) for 60 s. The under-etching of the LOR layer facilitated the lift-off process after metal deposition. A sputtering process was applied to deposit 200 nm of platinum with a 20-nm TiW adhesion layer (Ionfab 300, Oxford Instruments, Abingdon, UK). Lift-off was executed in Remover 1165 (Rohm-Haas, Schwalbach, Germany) using ultrasound. A 500-nm-thick Si_3_N_4_ passivation layer was then deposited onto the wafer using a plasma-enhanced chemical vapour deposition process (Plasmalab 80, Oxford Instruments). This passivation layer was re-opened at specified electrode sites using a patterned S1813 positive photoresist mask and reactive-ion etching (RIE; Plasmalab 100, Oxford Instruments).

Complementary rim structures and rings surrounding the electrodes on the sensor glass plug-in were fabricated using SU-8 resist. The 20-μm SU-8 rings were fabricated with SU-8 3025 (Microchem Corp.). The rim structures were fabricated with 2 layers of SU-8 100 following the same procedure described for the PDMS mould. After development, the glass wafer was diced into single sensor glass plug-ins (2.8 mm×13 mm) using a precision saw.

The individual glass plug-ins were glued onto a printed circuit board (PCB, 8 mm×91 mm×1.5 mm), wire bonded and packaged using epoxy glue (EPOTEK 302-3M, Dow Corning GmbH, Wiesbaden, Germany). Connector pins were soldered to the PCB to provide electrical connections.

### Electrode preparation

All sensor units were initially sterilized with 70% ethanol and isopropanol and cleaned with oxygen plasma for a duration of 3 min (Diener Electronic GmbH & Co.). The electrodes were tested by recording cyclic voltammograms (Compactstat, IVIUM Technologies, Eindhoven, The Netherlands). The electrodes were cycled in N_2_-bubbled, 1 M sulfuric acid solution between −0.25 and 1.60 V vs. Ag/AgCl at 100 mV s^−1^ at least 10 times. The reference electrode (RE) was coated with silver (Ag) via galvanostatic electrodeposition at −0.1 mA mm^−2^ for 300 s using an Ag wire as a combined counter/reference electrode; the silver on the RE was subsequently partially transformed into AgCl through the application of a constant current of 5 μA mm^−2^ for 1500 s in a 50-mM KCl solution. All four working electrodes (WEs) were coated with an m-polyphenylenediamine layer (mPPD; Sigma-Aldrich) using a 0.1 M mPPD solution in PBS. The polymer was deposited over 15 voltammetry cycles (0–0.9 V vs. Ag/AgCl). To improve the adhesion of the sensor hydrogel to the working electrodes, an additional (3-aminopropyl)triethoxysilane layer (Sigma-Aldrich) was deposited via vapour deposition for a duration of 2 h.

### Biosensor functionalization

The working electrodes were functionalized to detect glucose and L-lactate. Two different hydrogels, containing either glucose oxidase (GOx) or lactate oxidase (LOx), were prepared and deposited onto the electrodes under sterile conditions. The membrane hydrogel was prepared by adding the enzymes to an aqueous solution containing bovine serum albumin (BSA) and glutaraldehyde 25% (GA) to initiate cross-linking for hydrogel formation. For the glucose biosensor, 5 mg GOx (Sekisui Diagnostics, Kingshill, UK), 20 mg BSA (Sigma-Aldrich), 500 μl DI water, 10 μl Triton X-100 (3 g l^−1^) (AppliChem GmbH, Darmstadt, Germany), and 20 μl GA (Sigma-Aldrich) were used. For the L-lactate biosensor, 10 mg LOx (Sekisui Diagnostics, Kingshill, UK), 30 mg BSA, 500 μl deionized water, 10 μl Triton X-100 (3 g l^−1^), and 20 μl GA were used. An enzyme-free control hydrogel was formed using 30 mg BSA, 500 μl DI water, 10 μl Triton X-100 (3 g l^−1^), and 20 μl GA. The catalase membrane solution contained 1 mg catalase (Sigma-Aldrich), 30 mg BSA, 500 μl DI water, 10 μl Triton X-100 (3 g l^−1^), and 20 μl GA. GA was added at the end, after all other compounds had been thoroughly mixed for 15 min. The final solution was mixed for an additional 5 min using a magnetic stirrer^48^.

Sub-microlitre volumes of these solutions were manually transferred onto the electrodes by touching the small rim structure surrounding the electrodes with a 2-μl pipette using appropriate tips. The hydrogel was formed directly on the electrodes by curing the solution at room temperature for at least 3 h before use. For the preparation of multi-layer hydrogel electrodes, solutions were consecutively transferred to the electrodes using the same drop-coating technique, with 10 min of curing time in between.

### Device assembly

For the final assembly of a device, the sensor modules were inserted into the designated recesses on the microfluidic PDMS chip. Epoxy adhesive (Araldite, Huntsman, Everberg, Belgium) was poured between the microscopy slide hosting the PDMS structures and the PCB to fix and stabilize the device. As an alternative, double-sided adhesive tape could be used to reversibly affix the sensor module.

Prior to each experiment, the assembled device was activated by means of a 30-s oxygen plasma treatment (Diener Electronic GmbH & Co., Ebhausen, Germany), during which a thin PDMS mask with openings at the inlet, outlet, and all drop sites was used to cover all rim structures of the microfluidic hanging-drop device. This selective plasma treatment caused the inherently hydrophobic PDMS inside the circular drop structures and channel locations to enter a hydrophilic state. The covered rim structures remained hydrophobic, ensuring that the liquid would stay contained between them.

### Device operation

The assembled device was clamped upside down onto a custom-made chip holder and placed into an OmniTray cultivation box, covered with a lid; wet cotton pads were also placed in the box to provide additional humidity and minimize evaporation. The sensor unit was electronically connected to an in-house-fabricated CMOS-based multi-potentiostat for parallel current read-out^57^ and operated at a constant potential of 0.65 V between each working electrode and the on-chip RE. Precision syringe pumps (neMESYS, Cetoni GmbH, Korbussen, Germany) were used for flow control. The reported flow rates are given for single-drop rows. The device was placed in an incubation box with a controlled atmosphere of 37 °C, 5% CO_2_, and 95% humidity for optimal culture conditions. Inlet tubing was connected to a heatable perfusion cannula (PH01&TC02, Multichannel Systems, Reutlingen, Germany) to ensure a constant temperature.

### Sensor calibration

Off-chip calibrations were performed by immersing each functionalized biosensor unit in either 50 ml 1× phosphate-buffered saline (PBS) of pH 7.4 (Gibco, Life Technologies, Zug, Switzerland) or 50 ml RPMI 1640 cell culture medium (Chemie Brunschwig AG, Basel, Switzerland), including 100 IU ml^−1^ penicillin and 100 μg ml^−1^ streptomycin (Chemie Brunschwig). The temperature was set to 37 °C, and the liquid was constantly stirred using a magnetic stirrer. Defined volumes of prepared 100 mM D-glucose (Sigma-Aldrich) and 100 mM L-lactate (Sigma-Aldrich) solutions were added in a stepwise manner.

On-chip calibrations were performed using the same stock solutions. Lactate and glucose solutions were diluted to 0.25, 0.50, and 1.00 mM and loaded into glass syringes. The liquids were successively perfused through the device.

### Cell culture

All cell-based experiments were conducted using the fluorescent human colon carcinoma cell line HCT116 eGFP (Sirion Biotech, Munich, Germany). The cells were cultured in RPMI 1640 growth medium (Chemie Brunschwig) supplemented with 10% fetal bovine serum (FBS, Sigma-Aldrich), 100 IU ml^−1^ penicillin, 100 μg ml^−1^ streptomycin (Chemie Brunschwig), and 0.3 mg ml^−1^ puromycin (Sigma-Aldrich). The cell line was cultured in a humidified incubator (CB 60, Binder, Tuttlingen, Germany) at 37 °C, 5% CO_2_, and 95% humidity. When the cell culture reached a confluence of 80% in the 5-ml culture flasks, the cells were diluted with fresh medium and transferred to a new culture flask to ensure constant proliferation and long-term maintenance of the cell line.

Prior to an experiment, cells were collected from the culture flasks for microtissue fabrication. Old medium was removed from the cell culture flask, and the adherent cells were washed with 1× PBS of pH 7.4 (Gibco). The cells were treated with a 0.25% Trypsin-EDTA solution (Gibco) at 37 °C for 4 min to detach them from the culture plate. The cell suspension was diluted with fresh RPMI 1640 growth medium containing all supplements. The cell concentration was measured using a haemocytometer and adjusted with regard to the experimental requirements and desired microtissue sizes.

GravityPLUS plates (InSphero AG, Schlieren, Switzerland) were used to form and grow microtissues from HCT116 eGFP cell suspensions inside hanging drops. The cell concentrations were adjusted such that different hanging drops contained 250, 500, 1000, and 2000 cells each to modify the initial microtissue size. A multichannel pipette was used to transfer 40 μl of cell suspension to each hanging-drop site on the 96 GravityPLUS plate. After 3 days of incubation in the incubator at 37 °C, 5% CO_2_, and 95% humidity, microtissues of different sizes were formed and transferred to a GravityTRAP plate (InSphero) for maintenance and media exchange every 3 days.

### Measurement of analyte secretion

The glucose and lactate biosensors were calibrated to determine the specific sensitivity per area (nA mmol^−1^ L mm^−2^) of each electrode. During the experiments, the current density (nA mm^−2^) was constantly measured at a 10-Hz sampling rate. The corresponding analyte concentration was then calculated according to the previously determined sensitivity of the electrode in question. This procedure enabled continuous monitoring of the analyte concentration (mmol L^−1^) in the hanging drops over time.

Continuous measurements were performed as perfusion was periodically switched on or off, and the concentration changes in the 10-μl hanging-drop compartment were recorded in real-time (mmol L^−1^ h^−1^). The secretion rates (mmol h^−1^) were finally obtained by taking the single-drop volume of 10 μl into account.

## Results

### Device concept and design

**Figure 1:**
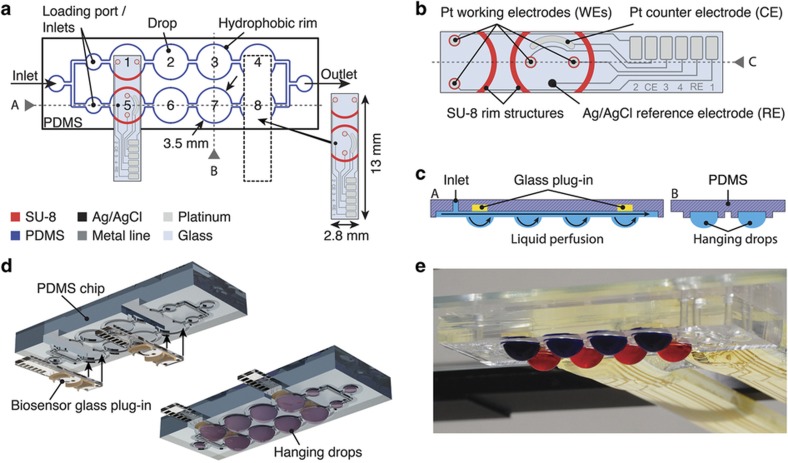
Design and assembly of the microfluidic hanging-drop network and sensor glass plug-ins. (**a**) Schematic two-dimentional (2D) top view of the microfluidic 2×4-hanging-drop chip showing the hydrophobic polydimethylsiloxan (PDMS) rim structures (blue). The dashed lines indicate a PDMS recess, into which a sensor plug-in is inserted. SU-8 structures on the glass plug-in complete the microfluidic network structures (red). (**b**) Close-up view of the sensor unit featuring four working electrodes (WEs), one counter electrode (CE), and one Ag/AgCl pseudo-reference electrode (RE), with the SU-8 rim structures indicated in red. (**c**) Cross section of the completely open microfluidic network showing the hanging drops, the inserted sensor glass plug-ins, and the perfusion flow. (**d**) Three-dimensional (3D) exploded view showing the device assembly (see Supplementary Figure S3 for an enlarged version). (**e**) Photograph of an assembled device loaded with colored liquid to better visualize the two rows of drops.

The biosensors are integrated into the hanging-drop network using a modular approach. The final device consists of two parts, a microfluidic PDMS chip and small glass plug-ins featuring the biosensor electrodes, as shown in Figure 1a. The hanging-drop network consists of eight circular regions, each with a diameter of 3.5 mm, at a pitch compatible with the 384-well-plate format (4.5 mm). The hanging drops form underneath these circular regions, which are interconnected by narrow channels. They are arranged in two rows of four drops, with three inlets and one outlet. In this configuration, medium can be guided in parallel through the microfluidic structures, allowing, for example, two different experimental conditions to be tested simultaneously. The PDMS substrate is biocompatible and transparent and offers the possibility to locally modify its wetting properties through O_2_-plasma treatment, such that hydrophilic surface regions surrounded by hydrophobic rim patterns can be realized. The rim structures stabilize the completely open microfluidic network.

The sensor plug-ins each extend over two drops and comprise six platinum electrodes and connection pads located on a 13 mm×2.8 mm glass substrate (Figure 1b). Two of these plug-ins can be inserted into one microfluidic PDMS chip. Two circular working electrodes (400 μm diameter) are integrated into each drop. Furthermore, a counter electrode and an Ag/AgCl pseudo-RE are integrated into one of the drops. SU-8 structures on the glass plug-in complete the rim patterns on the PDMS to obtain a leakage-free operation of the fluidic network. The sensor unit is inserted into a small rectangular recess in the PDMS chip such that the electrodes are located at the ceiling of the hanging drop in the final assembly (Figure 1c, cross section A, Supplementary Figure S4).

The device is loaded by pipetting 120 μl of liquid into one of the access holes, and eight hanging drops then form below the circular regions (Figure 1c, cross section B; Figure 1d). Each hanging drop has a volume of 10 μl, which includes the liquid in the cylindrical recess in the glass/PDMS substrate and the spherical drop fraction (Supplementary Figure S1). The remaining liquid volume is located in the channel and connection structures. The completely open PDMS chip architecture allows easy access to the fluidic system and facilitates the loading and harvesting of samples and spheroids during experiments.

**Figure 2:**
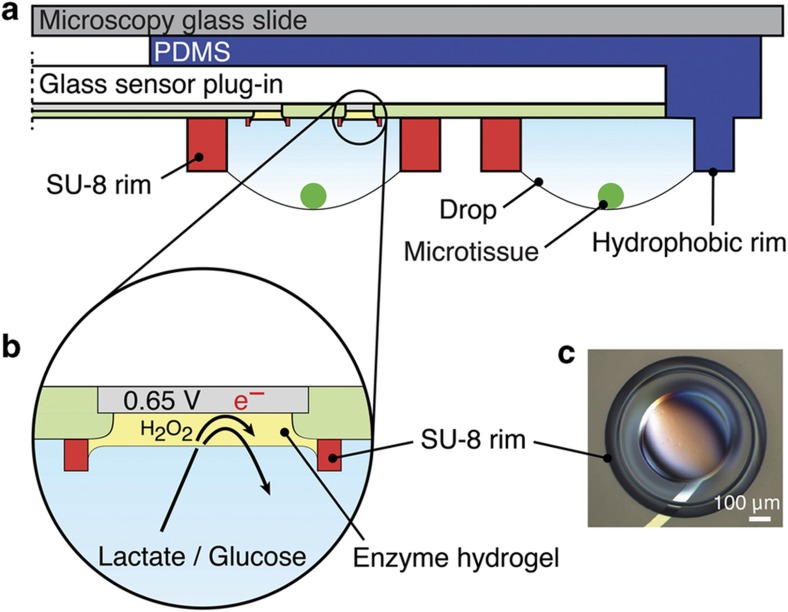
Configuration of the biosensor. (**a**) Schematic cross-sectional view of the biosensor inserted into the microfluidic hanging-drop network device (cross-section C in Figure 1b). Hanging drops form between the SU-8 rims on the glass substrate and the polydimethylsiloxan (PDMS) structure. The biosensors are located at the ceilings of the hanging drops. (**b**) Close-up view illustrating the working principle of the enzyme-based biosensors. Glucose oxidase (GOx) or lactate oxidase (LOx) transforms glucose or lactate to produce hydrogen peroxide, which is electrochemically detected on the Pt surface at 0.65 V vs. Ag/AgCl. (**c**) Photograph of a hydrogel-coated platinum working electrode showing the SU-8 ring structure, which facilitates precise hydrogel deposition.

A cross section of an assembled microfluidic hanging-drop device and sensor unit is shown in Figure 2a. The sensing electrodes allow monitoring of the medium around the microtissues in the corresponding drops. A simple drop-coating process was used to coat and functionalize the platinum working electrodes with a hydrogel containing immobilized glucose oxidase (GOx) or lactate oxidase (LOx). These enzymes catalyse an oxidation reaction of glucose or lactate that produces hydrogen peroxide, which, in turn, is amperometrically detected on the Pt electrodes at a potential of 0.65 V vs. Ag/AgCl. The current signal is directly correlated with the analyte concentration of interest (Figure 2b, reaction schemes in Supplementary Figure S5). A coated working electrode is shown in Figure 2c. The enzyme hydrogel is precisely located on the electrode area within the SU-8 ring structure. The ring structure facilitates the coating procedure and ensures uniform hydrogel deposition on all electrodes.

An important advantage of the modular approach is that the functionalization and calibration of the sensor can be performed independently and before plugging the sensor unit into the microfluidic PDMS substrate.

### Characterization of the glucose and lactate biosensors

**Figure 3:**
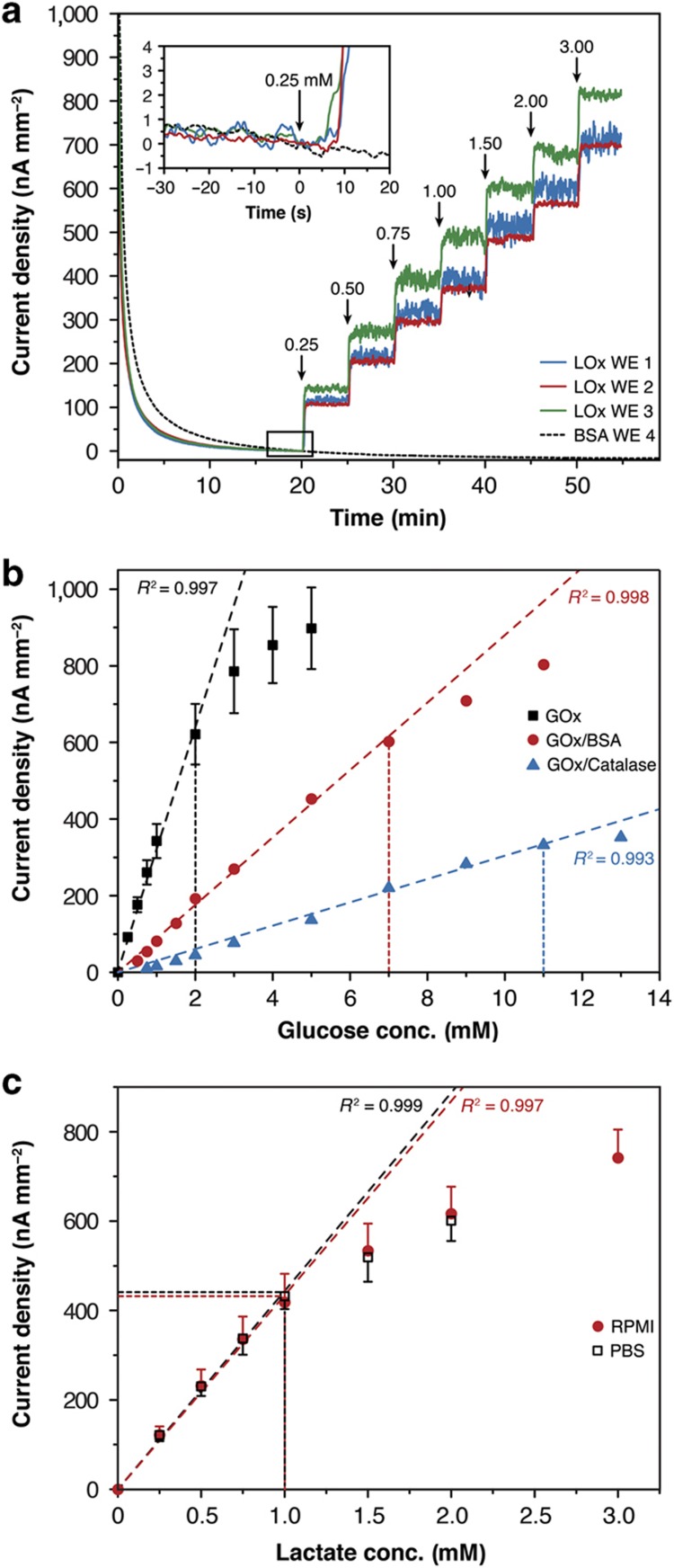
Off-chip calibration results for glucose and lactate biosensors at 37 °C for different biosensor compositions in various solutions. (**a**) Transient lactate current signals of three LOx-functionalized electrodes and one blank bovine serum albumin (BSA) electrode as a control in RPMI 1640 cell culture medium. The inset shows a zoomed-in view of the curves before the first lactate injection (the solutions were continuously stirred). (**b**) Glucose calibration curves showing the effects of different electrode functionalization protocols on sensitivity and linearity; an additional diffusion-limiting BSA hydrogel layer or a catalase membrane was applied (*n*=4 for glucose oxidase (GOx) coating; error bars represent standard deviation (s.d.)). (**c**) Lactate calibration in RPMI 1640 cell culture medium (*n*=3) and PBS (*n*=4; error bars represent s.d.).

The biosensors were characterized through multiple calibration experiments off-chip and were characterized on-chip after mounting in the microfluidic network. Raw data from an off-chip calibration of three functionalized LOx working electrodes and one blank BSA electrode in a conventional 50-ml beaker are shown in Figure 3a. After 20 min of settling at the beginning of the recording, lactate was added stepwise to the RPMI 1640 medium to introduce an increasing lactate concentration. A specific current response to lactate was recorded. The limit of detection (LoD) for the lactate biosensors was calculated to be 7.07±2.73 μM (three times the background noise at the end of the settling time) during off-chip calibration at 37 °C under continuous stirring.

The biosensors showed reproducible characteristics with regard to linearity, sensitivity, and reproducibility for both glucose and lactate (Figures 3b and c). A linear relationship was observed for up to 2 mM glucose for an electrode coated with a single GOx membrane (*R*^2^>0.99). This sensor exhibited a high sensitivity of 322±41 nA mM^−1^ mm^−2^. The biosensor characteristics could be tuned by implementing additional layers on top of the enzyme membrane. Adding, for example, a diffusion-limiting BSA layer on top of the enzyme hydrogel membrane increased the linear range up to 7 mM while decreasing the sensitivity to 88 nA mM^−1^ mm^−2^. Adding a catalase membrane on top of the enzyme membrane further increased the linear range to 11 mM. The sensitivity was determined to be 30 nA mM^−1^ mm^−2^. Figure 3c shows that the lactate sensor, with a simple LOx membrane coating on the electrode, showed similar linear response characteristics and sensitivity in RPMI 1640 cell culture medium (435±66 nA mM^−1^ mm^−2^) and PBS solution (443±37 nA mM^−1^ mm^−2^). The sensor exhibited a linear response for up to 1 mM lactate.

**Figure 4:**
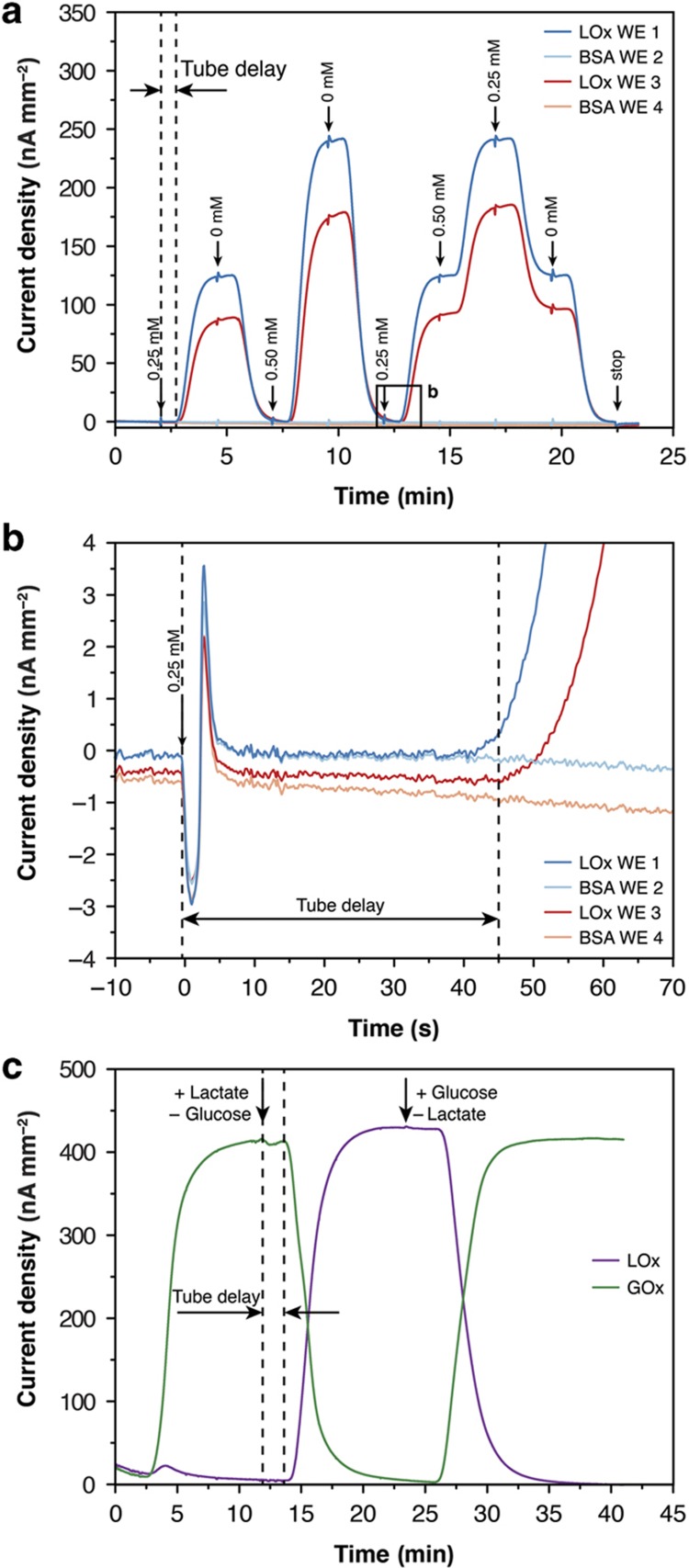
Sensor chip response curves for lactate and glucose in PBS at 37 °C. (**a**) Lactate signal recorded in a configuration featuring one lactate oxidase (LOx)-functionalized electrode and one blank BSA electrode per drop. The same liquid was infused in both rows in parallel at a flow rate of 10 μl min^−1^. The tube delay represents the time needed for the medium from the syringe to reach the hanging-drop instrumented with the sensor. The arrows indicate the instances when syringes were switched to introduce a different concentration/solution. (**b**) Close-up view showing the artifact observed upon syringe switching, the sensor response observed upon lactate reaching the hanging drop, and the low background noise levels of all four electrodes. The data have been offset-corrected to *t*=0 s. Negative current values can be attributed to incomplete settling at the beginning of the measurement. (**c**) On-chip lactate and glucose signals simultaneously recorded in phosphate-buffered saline (PBS) under an alternating analyte flow of glucose and lactate at a flow rate of 5 μl min^−1^.

The biosensors were then tested in the chip system in flow-through mode. Figure 4a displays raw signal traces simultaneously recorded by lactate biosensors and blank sensors during consecutive on-chip perfusion of 0, 0.25, and 0.50 mM lactate in PBS at a flow rate of 10 μl min^−1^. The response of the biosensors was selective and reversible. The two lactate sensors showed slightly different sensitivities, which can be attributed to variations in the manual membrane deposition process. These variations demonstrate the importance of performing calibrations before any biological measurement. For this characterization experiment, several syringe pumps supplying the various calibration solutions were connected to the device inlet via tubing. An artifact caused by the switching of the infusing syringe pump indicates a change in the calibration solution (Figure 4b). The presence of the tubing between the syringes and the microfluidic device produced a delayed sensor signal response. On the basis of the background noise, an LoD of 1.84±0.34 μM was calculated for this calibration.

Figure 4c presents raw signal traces recorded by a lactate and a glucose biosensor located in the same drop in response to consecutive perfusion of PBS containing 1 mM glucose and 1 mM lactate at a constant flow rate of 5 μl min^−1^. No sensor cross-talk was observed in the continuous-flow mode.

### Real-time online analyte recording

A typical characteristic of cancerous tissue is an enhanced glucose uptake rate due to an increased glycolysis metabolism, through which pyruvate is fermented to lactate under aerobic conditions^58^. Therefore, the performance of the device was assessed by measuring the metabolism of spherical green fluorescent protein (GFP)-induced human colon cancer microtissues (HCT116 eGFP). Glucose uptake and lactate secretion were recorded in real time in the microfluidic hanging-drop network using the two developed biosensors.

**Figure 5:**
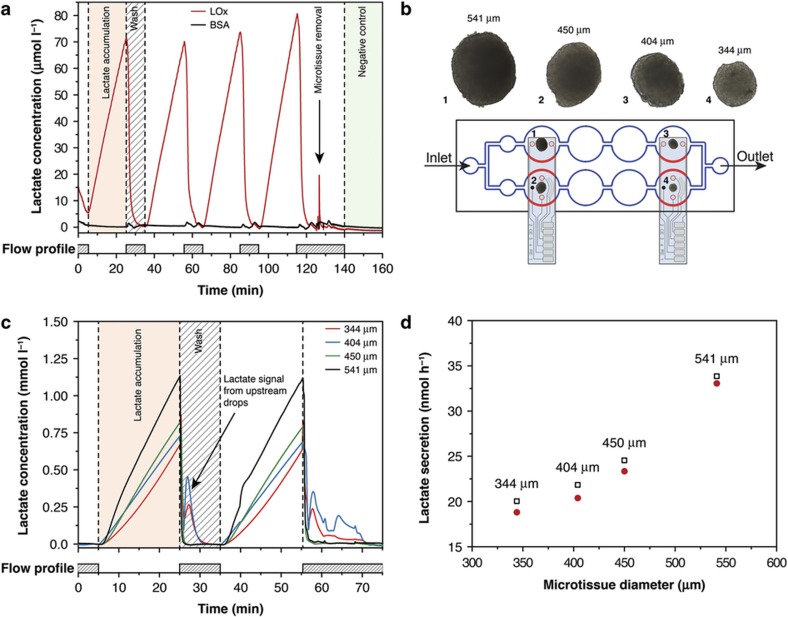
Measured lactate accumulation in hanging drops hosting HCT116 eGFP microtissues. (**a**) Lactate secretion from one microtissue sample cultured in phosphate-buffered saline (PBS) at 37 °C as measured during an on–off medium perfusion protocol, schematically depicted below the response curves. (**b**) Experimental arrangement and device set-up, with micrographs of the different HCT116 eGFP microtissue (not to scale) cultured in the different sensing drops in the hanging-drop network. The microtissues were located at the bottom of the hanging drops, at an approximate distance of 1 mm to the electrodes. (**c**) Lactate secretion of different microtissues cultured in RPMI 1640 cell culture medium at 37 °C during on–off perfusion (schematically depicted below the response curves). Carry-over signal peaks during the washing phase are clearly visible in the two downstream drops. (**d**) Microtissue-size-dependent lactate secretion rates (black boxes and red dots indicate secretion rates obtained from two consecutive measurements).

Figure 5a presents measurements of the lactate that was secreted from an HCT116 eGFP microtissue (480 μm in diameter) inserted into one of the sensor drops. The measurement was performed in PBS supplemented with 10 mM glucose while perfusion was stopped. After perfusion had been stopped, the lactate signal reproducibly increased to 73.8±4.4 μmol l^−1^ as a result of lactate accumulation in the drop and then decreased again during the washing phase, when the liquid volume in the drop was exchanged with fresh PBS at a flow rate of 5 μl min^−1^. A lactate secretion rate of 2.21 nmol h^−1^ was calculated. This value is comparable to the lactate secretion rates of growing HCT116 microtissues reported in the literature^59^. As a negative control, the microtissue was removed from the hanging drop after four perfusion cycles. No lactate was detected after removal, thereby confirming that the microtissue was the source of the lactate. Furthermore, no signal that might have originated from any other electroactive species was recorded on the blank electrode. A three-point calibration was performed prior to microtissue loading and after the experiment for the calculation of lactate concentrations.

In a second experiment, we investigated variations in the lactate secretion rate and their correlation with microtissue size. The experimental set-up and the microtissue arrangement are shown in Figure 5b. Two biosensors were inserted into the microfluidic network, and four HCT116 eGFP microtissues of different sizes were loaded into separate sensing drops. Figure 5c presents the raw signal curves of the lactate concentrations that were simultaneously recorded in all four hanging drops hosting the different microtissues. The following average lactate production rates were determined: 33.4 nmol h^−1^ for the largest, 541-μm-diameter microtissue; 23.9 nmol h^−1^ for the 450-μm-diameter microtissue; 21.1 nmol h^−1^ for the 404-μm-diameter microtissue; and 19.4 nmol h^−1^ for the smallest, 344-μm-diameter microtissue. The corresponding microtissue-size-dependent lactate secretion rates are plotted in Figure 5d.

Again, a washing step with fresh culture medium was performed by applying a perfusion flow of 5 μl min^−1^. The peaks observed in the signals from the sensors in drops 3 and 4 during the washing step are due to carry-over of the lactate and hydrogen peroxide that were produced and accumulated in the two sensing drops upstream of these drops in the microfluidic system. Similar peaks are not present in the sensor signals from drops 1 and 2.

**Figure 6:**
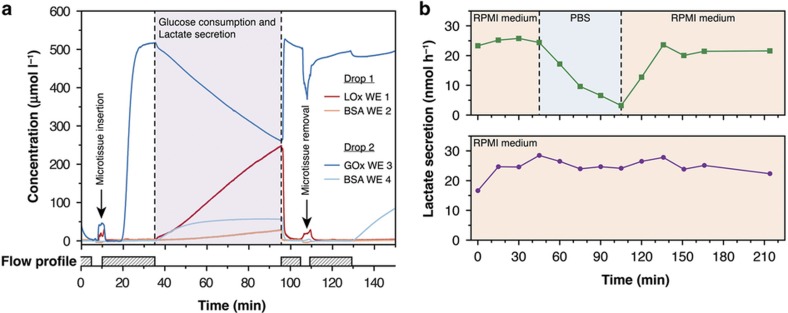
Monitoring the metabolism of HCT116 eGFP microtissues and their response to medium changes. (**a**) Glucose consumption and lactate secretion of HCT116 eGFP microtissues in phosphate-buffered saline (PBS) supplemented with 0.5 mM glucose at 37 °C under static culture conditions (sampling rate: 10 Hz). (**b**) The altered metabolism of a microtissue caused by the replacement of RPMI medium with glucose-free PBS in one of the drops and the recovery of lactate secretion after resupplementing with glucose (green curve). The other drop row on the same polydimethylsiloxan (PDMS) chip hosted a second microtissue (violet curve) and was continuously supplied with RPMI medium as a control.

Figure 6a presents *in situ* real-time measurements of the glucose consumption and simultaneous lactate production of four HCT116 eGFP microtissues of similar size (274±9 μm), which were inserted in pairs into two separate sensing drops. One drop contained a glucose sensor and a blank BSA electrode for glucose detection. In the other drop, the electrodes were functionalized to act as a lactate sensor and a blank BSA electrode. The measurement was performed by switching between PBS and glucose-supplemented PBS solution (0.5 mM) using an on–off perfusion protocol and a flow rate of 5 μl min^−1^. After microtissue insertion and the infusion of the glucose solution (clearly visible from the steep increase in the glucose signal), the microtissues were cultured for 60 min without flow. The glucose signal decreased from its initial value of 500 μM. The consumption rate for two microtissues was calculated to be 2.57 nmol h^−1^. Simultaneously, lactate was secreted at a rate of 2.40 nmol h^−1^. Comparable values for lactate secretion^59^ and glucose consumption^59,60^ have been reported in the literature. The blank BSA working electrodes showed slight cross-talk. This cross-talk signal originated from hydrogen peroxide diffusion in the same hanging drop. The liquid was exchanged after 1 h, restoring the initial sensor signal values for both sensor types. The microtissues were then removed from both sensing-drop sites. Perfusion was stopped again, and subsequent measurements confirmed that the recorded analyte transients indeed originated from the metabolism of the microtissues.

The developed microfluidic PDMS chip features separate inlet holes for each drop row (Figure 1a). These inlets can be used to perfuse different solutions in parallel. This configuration permits direct comparison of the effects of medium variations or the different dosages of compounds on the metabolic activity of cultured microtissues. Figure 6b shows the lactate secretion from HCT116 eGFP microtissues (553 and 566 μm) observed while changing from RPMI medium to glucose-free PBS in one of the two drop rows (green curve). The lactate secretion rate of the microtissue immediately began to decrease from its initial value of 22.5–25.0 to 3.0 nmol h^−1^, whereas no change in the secretion rate was observed for the control microtissue that was continuously cultured in RPMI (violet curve). Switching the flow back to RPMI stimulated the lactate secretion to nearly the initial rate of approximately 20.0–22.5 nmol h^−1^. The lactate measurements were performed by applying an on–off perfusion protocol (similar to previous experiments) that consisted of measuring the lactate secretion over 10 min under static conditions and then perfusing fresh medium for 5 min at 2.5 μl min^−1^.

## Conclusion

We introduced an analytical platform for the real-time *in situ* multi-analyte monitoring of 3D microtissue spheroids by integrating sensor features into a microfluidic culture device. The implementation is based on a modular approach, in which glucose and lactate multi-electrode biosensor modules can be inserted as plug-ins into a microfluidic hanging-drop network. Through this approach, all advantages of the hanging-drop network technology can be fully exploited, including the adhesion-free liquid–air interface for spheroid cultures, precise control of the liquid flow, bubble-free operation, inherent gas exchange, and full continuous access to the fluidic network for the loading and collecting of liquid samples and spheroids for downstream analysis.

The decoupling of the sensor unit from the microfluidic chip substantially reduces the fabrication, assembly, and operational complexity of the integrated multi-functional platform. Critical procedures, such as biosensor functionalization and calibration, can be performed independently off-chip. Biosensors can, for example, be prepared and stored and then plugged in immediately before use, when the microtissue structures are ready for experimentation. The developed functionalization procedure is simple, and its reproducibility has been improved through the implementation of SU-8 electrode rims that support drop deposition.

The lactate and glucose metabolism of individual GFP-induced human colon carcinoma microtissues was successfully measured *in situ* inside small, 10-μl volumes using an automated perfusion set-up, such that any disturbance from or influence of external effects could be effectively excluded. The obtained data provided real-time information on a time scale of minutes regarding the metabolic state of the microtissues under different culture conditions.

This technological approach is highly versatile. Different versions of the microfluidic chip were fabricated, in which the biosensor modules could be plugged in at different locations in the hanging-drop network. The approach can be applied in larger and more complex hanging-drop networks including different spheroid types in multi-tissue configurations. Furthermore, the read-out capability of the system can be easily expanded through the integration of additional plug-ins comprising more and/or tissue-specific sensor types. The platform is very flexible in how the sensors and the hanging drops hosting the microtissues can be arranged.

Although the presented approach allows continuous sensor operation at good sensitivity for ~1 day, biosensors always face challenges related to extending their lifetimes and achieving good measurement reproducibility. We attempted to address this challenge by using the plug-in approach, which allows sensors with satisfactory characteristics to be selected and permits pre- and post-calibration to be performed off-chip or by using an automated on-chip perfusion protocol during longer experiments. Thus, a variety of biological questions can be addressed using the presented method; for experiments extending over longer time spans, optimization of the sensors with respect to long-term stability will be required.

With the presented design for biosensor integration, we have introduced an important feature into the hanging-drop network technology. The proposed sensors enhance the data and information that can be collected from such systems and enable new insight into multi-tissue or ‘body-on-a-chip’ experiments.
