# Supplementary material for: Multi-analyte biosensor interface for real-time monitoring of 3D microtissue spheroids in hanging-drop networks
Source: Microsyst Nanoeng. 2016 Jun 6;2:16022. doi: 10.1038/micronano.2016.22 (PMC6444714; doi:10.1038/micronano.2016.22)
Supplement: Supplementary Information [file micronano201622-s1.pdf]

Supplementary file

Multi-analyte biosensor interface for real-time monitoring of 3D microtissue spheroids in hanging-drop networks

Patrick M. Misun, Jörg Rothe, Yannick R.F. Schmid, Andreas Hierlemann and Olivier Frey

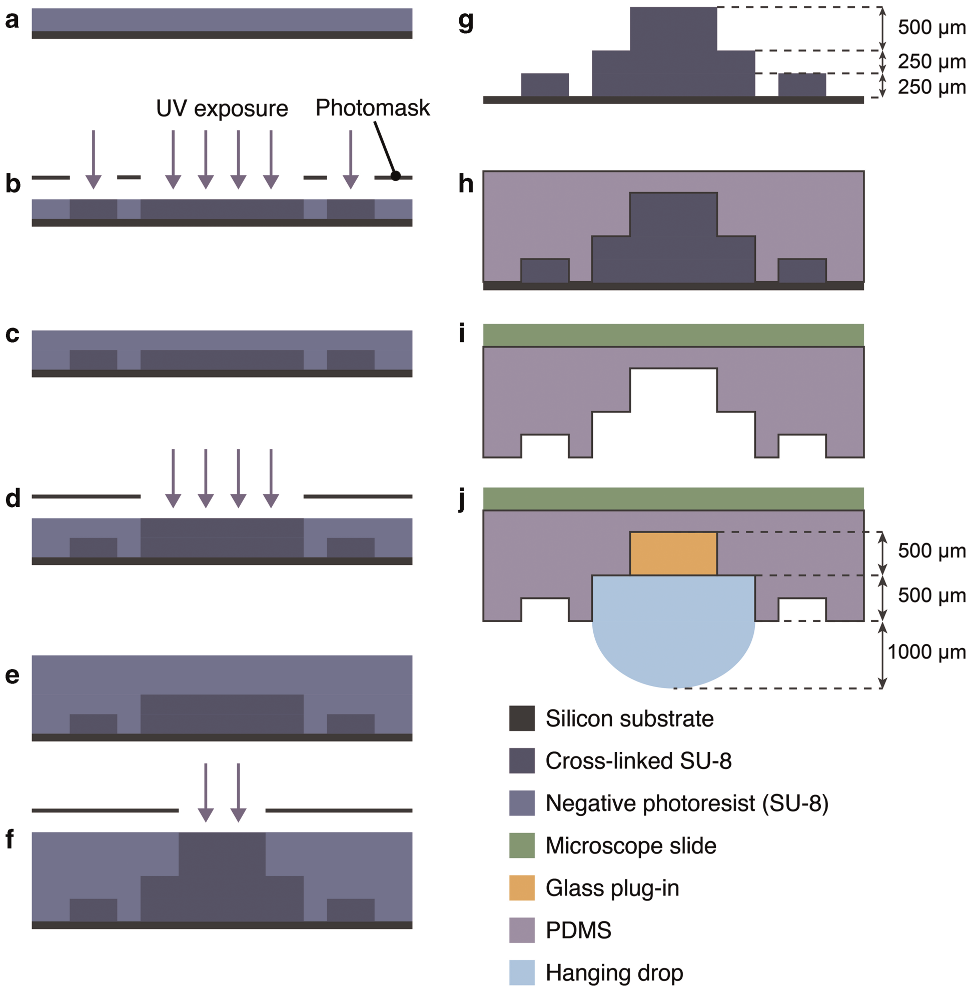

Figure S1 Fabrication of the SU-8 master mold for casting the microfluidic hanging-drop network. (**a**) Spin-coating of the first SU-8 layer (250 µm) on the silicon substrate. (**b**) UV exposure and patterning through a transparency mask and cross-linking of exposed SU-8. (**c**–**f**) Repeated process for SU-8 layers 2 (250 µm) and 3 (500 µm). (**g**) Development of non-crosslinked SU-8 of all layers. (**h**) PDMS casting and curing. (**i**) Bonding of PDMS onto a glass slide to improve stability. (**j**) Insertion of sensor glass plug-in and liquid loading.

ETH Zürich, Department of Biosystems Science and Engineering, Bio Engineering Laboratory, Mattenstrasse 26, Basel CH-4058, Switzerland.
Correspondence: Patrick M. Misun (patrick.misun@bsse.ethz.ch) or Olivier Frey (olivier.frey@bsse.ethz.ch)

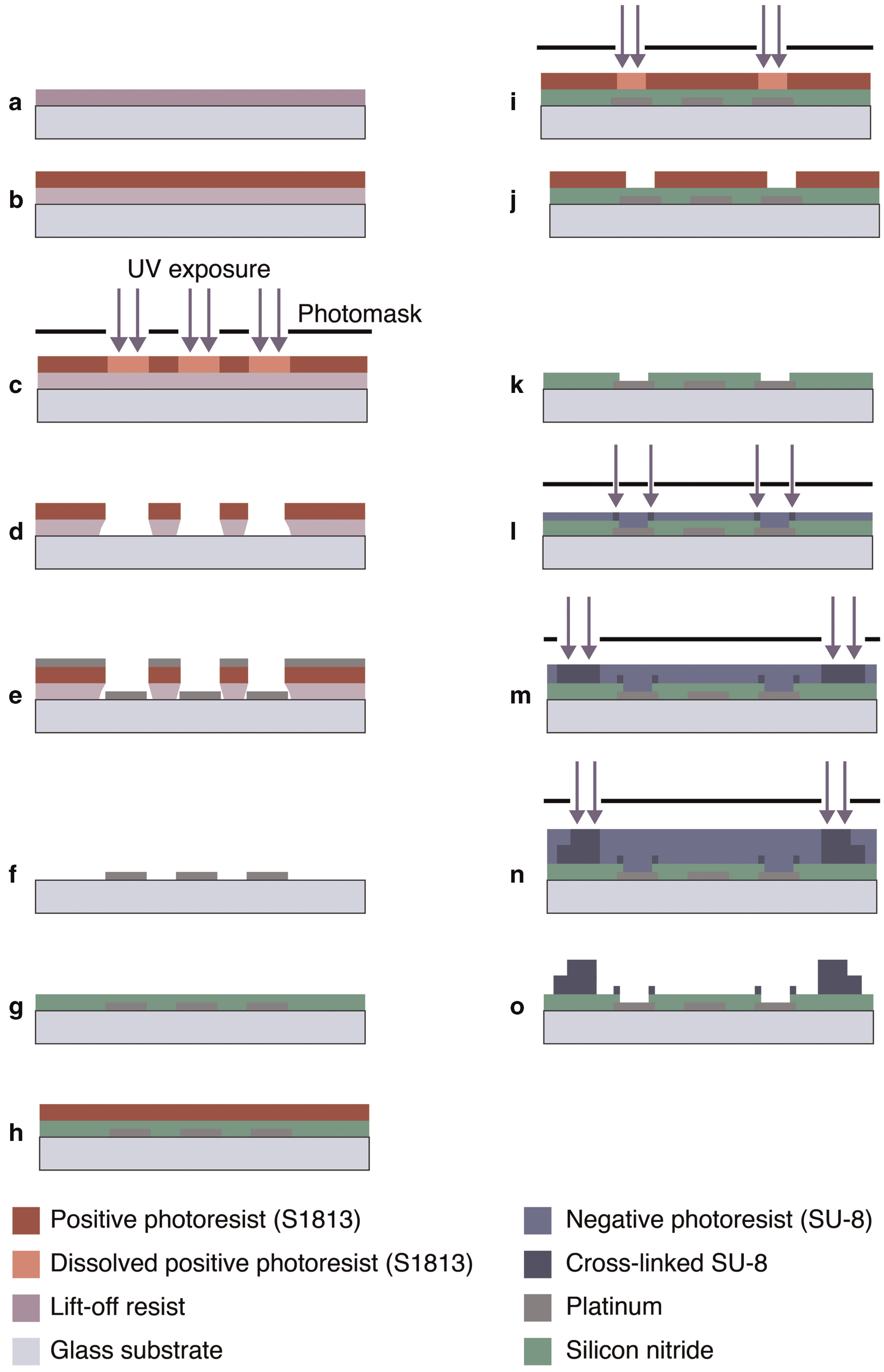

Figure S2 Fabrication of the sensor glass plug-in. (**a**) Spin-coating of the lift-off resist on the glass substrate. (**b**) Subsequent spin-coating of positive photoresist (S1813). (**c**) UV exposure through a transparency mask for metal patterning. (**d**) Development of photoresist and lift-off resist (exposed photoresist is dissolved) (**e**) Sputtering of the metal layer (20 nm TiW, 200 nm Pt). (**f**) Lift-off. (**g**) Plasma-enhanced vapor deposition of silicon nitride for passivation. (**h** and **i**) Spin-coating of positive photoresist (S1813) and UV exposure through a transparency mask. (**j**) Development of the exposed resist. (**k**) Reactive-ion etching to open platinum electrode sites. Remaining photoresist is removed by oxygen plasma. (**l**–**n**) Consecutive spin-coating of three SU-8 layers (20, 250, and 250 μm), and UV exposure through transparency masks. (**o**) Development of all SU-8 structures.

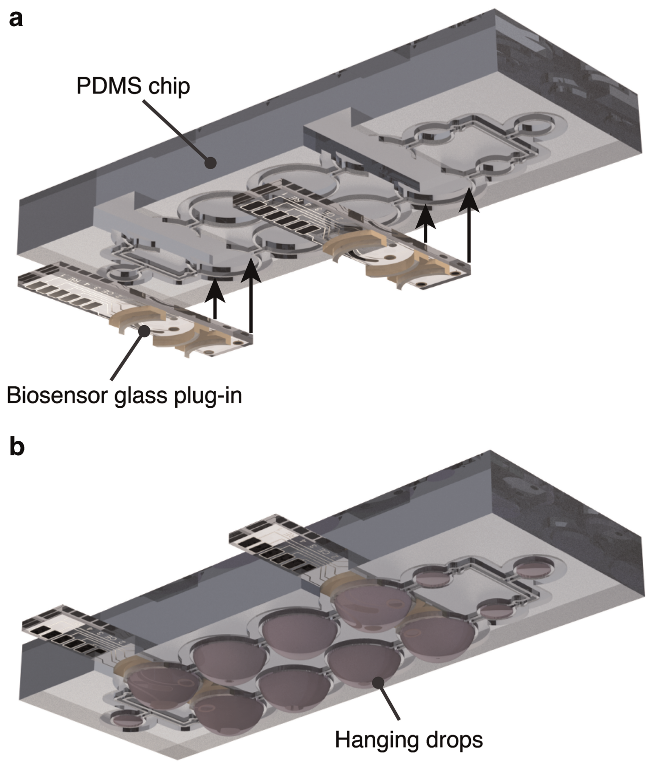

Figure S3 3D exploded view showing the device assembly. (**a**) Insertion of sensor glass plug-ins into a microfluidic PDMS hanging drop network (PCB is not shown). (**b**) Hanging drops are formed underneath the PDMS chip after liquid loading so that analyte measurements can be performed on four drops in parallel.

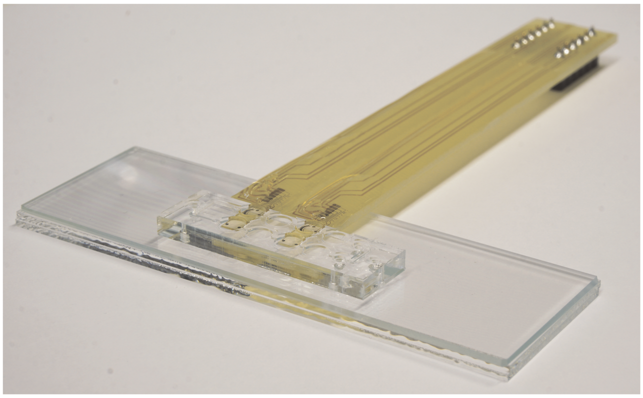

Figure S4 2 × 4 Hanging-drop network device with two sensor modules inserted into the microfluidic PDMS chip before liquid loading. The sensor glass plug-ins are glued to a small PCB and wire-bonded to connector pins at the end of the PCB to facilitate electric connection. Different PDMS chip layouts were designed, which allowed for inserting one or two identical sensor modules in parallel into different drops (in this image in columns 1 and 3).

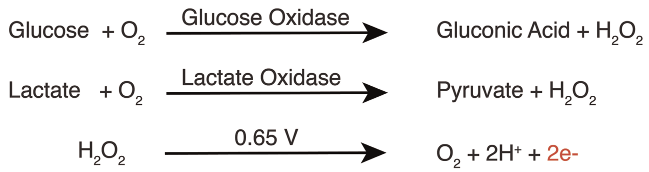

Figure S5 Analyte detection principle and reaction scheme of the sensor. Glucose oxidase (GOx) and lactate oxidase (LOx) catalyze an oxidation reaction of the analytes, i.e., glucose and lactate react to gluconic acid and pyruvate in the presence of oxygen. Hydrogen peroxide is produced as a side product and is oxidized on the electrodes at a potential of 0.65 V vs. Ag/AgCl. This oxidation process can be quantitatively monitored by amperometry, and the obtained signals directly correlate with the analyte concentration.
